# A naturally occurring promoter variation of *BrGSL-OHa* contributes to the conversion of gluconapin to progoitrin in *Brassica rapa* L. leaves

**DOI:** 10.3389/fpls.2025.1654238

**Published:** 2025-09-24

**Authors:** Jingyi Zheng, Su Ryun Choi, Yue Jing, Wenjun Zhang, Yan Sun, Xiaonan Li, Yong Pyo Lim

**Affiliations:** ^1^ Molecular Biology of Vegetable Laboratory, College of Horticulture, Shenyang Agricultural University, Shenyang, China; ^2^ Molecular Genetics and Genomics Lab, Department of Horticulture, Chungnam National University, Daejeon, Republic of Korea; ^3^ Dalian Modern Agriculture Development Service Center, Dalian, China

**Keywords:** *Brassica rapa*, glucosinolates, QTL, BrGSL-OHa, BrMYB29b

## Abstract

Glucosinolates (GSLs) are sulfur-rich secondary metabolites that play important roles in human health, plant defenses against pathogens and insects, and flavor. The genetic architecture of GSL biosynthesis in *Brassica rapa* L. remains poorly understood despite several mapping and gene prediction studies. This study conducted a conventional quantitative trait locus (QTL) analysis to identify putative genes regulating GSL biosynthesis in *B. rapa* in two field trials. Four consensus QTL clusters were identified for various GSL compounds. Six QTLs exhibited effects of QTL–environment interactions (Q×E), reflecting the genetic variation underlying phenotypic plasticity. QTL-Cluster2 and QTL-Cluster3 on chromosome A03 represented two genetically stable regions for major aliphatic GSLs (Ali-GSLs) without Q×E effects. Interestingly, variation in the expression of *BrGSL-OHa*, rather than gene sequence variation, explained the association between QTL-Cluster2 and gluconapin and progoitrin accumulation in *B. rapa*. Further function analysis indicated that the lack of an MYB binding site in the oil-type *B. rapa BrGSL-OHa* promoter region represented a rare non-functional allele among *B. rapa* genotypes, which prevented binding with the MYB transcription factor BrMYB29b, thereby repressing *BrGSL-OHa* transcription and inhibiting progoitrin biosynthesis. This study provides new insights regarding the molecular regulatory mechanism of GSL biosynthesis in *B. rapa*.

## 1 Introduction

Glucosinolates (GSLs), a class of sulfur-rich secondary metabolites largely limited to the Brassicaceae crops, are known to play important roles in disease resistance, insecticide metabolism, human health, and flavor quality (Wittstock and Burow, 2001; Soundararajan and Kim, 2018). Extensive variation in GSL profiles has been detected in *Brassica* crops, such as pakchoi (*Brassica rapa*), Chinese cabbage (*Brassica rapa* L.), broccoli (*Brassica oleracea*), cabbage (*Brassica oleracea* L.), and oilseed rape (*Brassica napus* L.), as well as *Arabidopsis* (Brachi et al., 2015; Zhu et al., 2022). GSLs are hydrolyzed by myrosinase to form degradation products with diverse biological activities, such as isothiocyanates, nitrile, thiocyanates, and oxazo-lidine-2-thione (Fahey et al., 2001). GSLs were initially thought to be toxic based on the 2-hydroxy-3-butenyl GSL progoitrin (PRO), which causes goiter in mammals (Kloss et al., 1996; Smolinska et al., 1997). However, the beneficial effect of 4-methylsulfinylbutyl GSL (glucoraphanin) has since been recognized due to the anti-carcinogenic activity of its degradation product (Sharma et al., 2011).

A suite of crucial structural and transcription factor genes involved in the GSL biosynthesis pathway have been identified in *Arabidopsis*. Genetic and functional analyses have revealed the important roles of the methylthioalkylmalate synthase (*MAM*) and 2-oxoglutarate-dependent dioxygenase (*AOP*) gene families in the side-chain elongation and modification of aliphatic GSLs (Ali-GSLs) (Kliebenstein et al., 2001a, b; Kroymann et al., 2001). In previous research, the *GSL-OH* locus was cloned and found to be a 2-oxoacid-dependent dioxygenase (2ODD) involved in the formation of PRO from the precursor gluconapin (GNA) (Hansen et al., 2008). Moreover, several R2R3-MYB transcription factors, namely, MYB28, MYB29, MYB76, MYB51, MYB34, and MYB122 have been identified as regulators for Ali-GSLs and indolic GSLs (Ind-GSLs) (Gigolashvili et al., 2007a, b; Hirai et al., 2007; Gigolashvili et al., 2008; Frerigmann and Gigolashvili, 2014). Homologs of these Arabidopsis MYB genes have been identified and characterized in various Brassica species, including *B. napus*, *B. oleracea*, and *B. rapa*, where they similarly orchestrate the regulation of GSL biosynthesis, albeit with increased complexity due to genome polyploidization (Liu et al., 2020; Zuluaga et al., 2019; Seo et al., 2016).

In *Brassica* crops, conventional quantitative trait loci (QTLs) or expression QTLs (eQTLs) are valuable approaches for dissecting the genetics of GSL synthesis. A meta-QTL analysis of GSL biosynthesis in different organs of *B. oleracea* uncovered the central role of the epistatic interaction of the alkenyl GSL locus (GSL-ALK) with other QTL loci (Sotelo et al., 2014). The candidate genes *BoGSL-Elong* (*MAM*) and *BoGSL-ALK* (*AOP*) have been cloned and characterized in *B. oleracea* (Li and Quiros, 2002, 2003). Hirani et al. (2013) developed *B. rapa* seeds low in 5C Ali-GSLs through replacing the A-genome functional locus with the *B. oleracea* non-functional *GSL-ELONG* locus. In *B. napus* and *B. juncea*, QTL and genome-wide association study (GWAS) analyses of GSL content have focused on seeds and several candidate genes have been identified, including *GTR2a*, *MYB28*, *GSH2*, *MAM1*, and *AOP2* (Ramchiary et al., 2007; Bisht et al., 2009; Feng et al., 2012; Harper et al., 2012; Li et al., 2014; Lu et al., 2014; Rout et al., 2015; Kittipol et al., 2019; Liu et al., 2020; Yang et al., 2021). Until recently, limited studies have reported the genetic dissection of QTLs controlling GSL accumulation in *B. rapa*. Lou et al. (2008) first identified 16 loci controlling the accumulation of Ali-GSLs, three loci for Ind-GSL accumulation, and three loci for aromatic GSL (Aro-GSL) accumulation. Using the same parental materials as those described by Lou et al. (2008), a doubled haploid segregation population was developed and both the metabolism QTLs and eQTLs were analyzed to elucidate the regulatory network of the GSL synthesis pathway (Pino Del Carpio et al., 2014).

Following the assembly of a whole-genome sequence for *B. rapa*, 102 putative GSL genes orthologous to 52 *Arabidopsis* GSL genes were identified in the *B. rapa* genome (Wang et al., 2011a). Most *B. rapa* GSL genes were present as two or three copies (Zang et al., 2009; Wang et al., 2011a). The genome-triplication event in the ancestor of *B. rapa* accounts for the greater complexity of the GSL metabolic pathway in *B. rapa* compared to *Arabidopsis*. Currently, the functional association of GSL-related genes with GSL accumulation in *B. rapa*, and the mechanisms responsible for the substantial variation in GSL profiles and contents among genotypes is poorly understood. Thus, it is necessary to identify functional GSL-related genes in large-scale and their underlying molecular genetic mechanisms.

This study aimed to identify and verify potential candidate genes associated with GSLs in *B. rapa* and their regulatory mechanisms. To unravel the genetic architecture of GSL biosynthesis and identify alleles underlying the natural variation in GSLs, conventional QTL analysis was performed using a bi-parental mapping population of *B. rapa* over 2 years. The results of the present study enhance the understanding of the genetic architecture of complex GSL metabolism and will contribute to the genetic improvement of GSLs in *B. rapa*.

## 2 Material and methods

### 2.1 Bi-parental mapping population

To map QTLs that control leaf GSL compounds, this study employed a biparental mapping population, CRF_2/3,_ comprising 190 individuals derived from a cross between ‘Chiifu 401-42’ (hereafter Chiifu), a heading Chinese cabbage used as the reference material for the *B. rapa* genome sequencing project, and rapid-cycling *B. rapa* (RCBr) (Li et al., 2010, 2013). Two independent experiments were conducted to detect the leaf GSL content in the CRF_3_ population. In experiment I (2012SG), both parental lines and the CRF_3_ population were grown in pots from March–July 2012 in a glasshouse under a 16-h/8-h (light/dark) photoperiod at 24 °C in Daejeon, Korea. One fully expanded leaf from each of two plants per F_3_ family line was harvested after 40 days of growth. A second experiment (2013AF) was performed in an open field at Chungnam National University, Daejeon, Korea, from August–November 2013. Fully expanded leaves were taken and mixed from three plants per F_3_ family line at the four-to-five leaf stage. Leaf samples were stored at −80 °C for subsequent freeze-drying and GSL extraction.

### 2.2 Diverse *B. rapa* genetic lines

A diverse panel consisting of 85 *B. rapa* inbred or double-haploid lines, including heading Chinese cabbage, pakchoi, oil-type, and caixin lines, was employed for GSL compound detection and *BrGSL-OHa* gene sequence analysis (**Supplementary Table S1**). Seeds for each *B. rapa* line were germinated in cell trays in a glasshouse for 1 month and then transferred to an open field at Shenyang Agricultural University, China and grown from August–December in 2016. Twelve plants per line were arranged in a randomized block design with two replications. At the harvest stage, for heading Chinese cabbage lines, outer leaves were sampled and mixed with the heading leaves. For the non-heading types, two to three mature leaves were collected. For each replication, leaf samples from six plants per fixed line were collected and mixed for subsequent GSL analysis.

### 2.3 Glucosinolate isolation and quantification

Freeze-dried leaf samples were ground into powder using a domestic mixer, and 0.1 g of sample powder was utilized for GSL extraction with 70% methanol following the method described by Kim et al. (2013). Extraction solution desolated by sulfatase was loaded onto an ion exchange column filled by Sephadex DEAE-A25, Sigma. A high-performance liquid chromatography (HPLC) instrument equipped with an ultraviolet detector was employed for chromatographic analysis. The HPLC column was a C18 Atlantis water column with a particle size of 2.5 µm and a length of 150 mm. GSL compounds were detected at 229 nm and separated according to the following method: in 2% acetonitrile for 0–5 min; a 15-min gradient from 0% to 25%; and a final 10 min in 2% acetonitrile. The flow rate was 1.0 mL min^−1^. For mass spectrometry analysis, the eluate was directed to a quadrupole mass spectrometer equipped with a positive electrospray ionization source. The spray voltage was set to 4.5 kV and the capillary temperature was maintained at 250 °C. Mass scanning was performed over a range of 100 to 700 m/z, according to the method described by Kim et al. (2013). GSLs were quantified using sinigrin (SIN, sinigrin monohydrate; Sigma-Aldrich) as an external standard. Concentrations are expressed as µmol per gram of dry weight (µmol·g^-^¹ DW).

### 2.4 Statistical and QTL analyses

The genetic map utilized for the QTL analysis of GSL compounds was updated by anchoring a small number of GSL gene-specific single-nucleotide polymorphism (SNP) markers onto a previously constructed genetic map sketch using the CRF_2_ mapping population. Putative GSL-related ortholog genes in the *B. rapa* genome were detected according to a comparative genome study by Wang et al. (2011a) and are listed in **Supplementary Table S2**. Information on the GSL gene-specific SNP markers is presented in **Supplementary Table S3**. Analysis of variance (ANOVA) between the replications of two experimental trials was conducted using GenStat.

WinQTL Cartographer version 2.5 was employed to perform QTL analysis using the composite interval mapping (CIM) function as previously described (Li et al., 2013) utilizing single-environment phenotypic data. Tests for the presence of QTLs were performed at 2-cM intervals using a window of 10 cM. Significant QTL-defining LOD (Logarithm of the odds) values were calculated with 1000 permutations for each GSL phenotypic dataset obtained in 2012 and 2013. To assess the QTL–environment interaction, a multi-environment approach (MEA) for individual GSLs was implemented following the mixed-model technique described by Boer et al. (2007). The first step was to select the variance–covariance (VCOV) model best that suited the phenotypic data across two environments. The VCOV model was employed to model the variation between genotypes both across and within environments. The entire genome was scanned using simple interval mapping and the candidate QTLs were selected as co-factors to perform a second-round composite interval mapping (CIM) scan. Finally, QTL backward selection for loci in multiple environment trails was determined and the allelic effects of QTLs in each environment and the QTL x E effect were estimated. To distinguish the QTL results obtained using this two-analysis method, a capital “E” was added following the names of the QTLs obtained using the MEA.

### 2.5 Expression analysis of candidate genes using quantitative real-time polymerase chain reaction

To confirm the expression level of the candidate gene *BrGSL-OHa* in the QTL-cluster region, the total RNA was extracted from 45- and 90-day-old leaf samples collected from the parental lines Chiifu and RCBr as well as selected fixed lines using the RNA-prep Pure Plant Total RNA Extraction Kit (Tiangen, Beijing, China). The expression levels of GSL biosynthesis-related genes in wild-type and OE-*BrMYB29b* transgenic Chinese cabbage lines were detected. cDNA was synthesized from 1 µg RNA for semi-quantitative and qRT-PCR in accordance with the method of Zheng et al. (2025). Internal standards using the *B. rapa* actin gene and gene-specific primer pairs are displayed in **Supplementary Table S4**.

### 2.6 Identification and analysis of *BrGSL-OHa* promoter sequences among different *B. rapa* genetic lines

To clone the *BrGSL-OHa* promoter sequences of different *B. rapa* materials, primers were designed according to the −1.5–2.0 kb upstream sequences of *BrGSL-OHa* obtained from the Chiifu reference genome. The forward primer was designed in the 5’-upstream sequence of *BrGSL-OHa* and the reverse primer was designed in the *BrGSL-OHa* coding sequence (**Supplementary Table S4**). DNA was isolated from the leaves of 85 *B. rapa* genetic lines (**Supplementary Table S1**). PCR was performed in a 50-ul reaction mixture containing 2 ul DNA template, 0.2 uM of each primer, and 25 ul of 2 Ex Taq Mix (Takara, Dalian, China). The PCR products were cloned into T-Vector pMD™20 (Takara, Dalian, China) for sequencing. The online tool PlantCARE (http://bioinformatics.psb.ugent.be/webtools/plantcare/html/) was employed for the prediction of cis-acting regulatory elements.

### 2.7 Heterologous expression and enzyme assays for BrGSL-OHa

The full-length cDNA of *BrGSL-OHa* was amplified from Chiifu and cloned into the pET30a expression vector. The constructed plasmid was expressed in *Escherichia coli* strain BL21(DE3) grown in LB (Luria-Bertan) medium to an OD_600_ value at 0.6. The induction of recombinant protein synthesis was initiated by adding 0.2 mM isopropyl β-d-thiogalactopyranoside overnight at 15 °C. The expressed cells were sonicated with 50 mM Tris (pH 8.0), 300 mM NaCl, 20 mM imidazole containing 1% Triton X-100, 1 mM DTT, and 1 mM PMSF. At the same time, the Ni-IDA affinity chromatography column was balanced with 50 mM Tris (pH 8.0), 300 mM NaCl, and 20 mM imidazole buffer. The target protein was then eluted with different concentrations of imidazole balanced buffer, and each elution component was collected for SDS-PAGE analysis. Enzyme assays were conducted as described by Zhang et al. (2015) using purified GNA as substrate. Liquid chromatography–electrospray ionization mass spectrometry was employed to detect GSL profiles (Waters ACQUITY UPLC H-Class_XEVO TQD). The mass spectrometer was operated in the positive electrospray ionization mode using the following manual optimization of the general conditions: capillary voltage: 2000 V; cone voltage: 14 V; desolvation gas flow: 1000 L/h; desolvation temperature: 650°C; scanning mode: daughter scan; collision energy: 10 V; fragment scan range: 50–330 m/z; scan time: 0.3 s.

### 2.8 Gene cloning, vector construction, and plant transformation

For the overexpression (OE) construct, the 1.14-kb coding sequence of *BrGSL-OHa* was amplified from Chiifu cDNA and cloned into the vector pBWA(V)HS driven by the cauliflower mosaic virus (CaMV) 35S promoter (35S_pro_: *BrGSL-OHa* ^chiifu^). To test the effect of two promoter types on PRO synthesis, two constructs were generated. The 3.02-kb sequence fused by the 1.88-kb promoter and the 1.14-kb coding sequence of *BrGSL-OHa* obtained from Chiifu was amplified and cloned into the vector pBWA(V)HS to generate construct I (*BrGSL-OHa* ^chiifupro:^ *BrGSL-OHa* ^chiifu^). The 2.97-kb sequence that included the 1.83-kb promoter and the 1.14-kb coding sequence obtained from oil-type *B. rapa* RCBr was utilized to generate construct II (*BrGSL-OHa* ^RCBrpro:^ *BrGSL-OHa* ^RCBr^). Wild-type control plants were generated by introducing the empty vector. Chinese cabbage is quite recalcitrant to genetic transformation and is dependent on genotypes. Explants obtained from the Chinese cabbage inbred line C-24 developed in our previous study (Li et al., 2021; Zhang et al., 2025), which exhibits relatively high genetic transformation efficiency, were utilized for OE construct transformation to validate *BrGSL-OHa* gene function. Because C-24 was difficult to utilize in resistant shoot formation when transforming constructs I and II, the constructs with combinations of the *BrGSL-OHa* promoter and genes were transformed into Westar, an oilseed genotype with low GSL contents and high transformation efficiency, using the *Agrobacterium tumefaciens* EHA105-mediated method via cotyledon explants.

To further explore the regulatory role of transcription factor *BrMYB29b* in PRO biosynthesis, the full-length cDNA of *BrMYB29b* from Chiifu was cloned into pBWA(V)HS to construct the OE vector. The *BrMYB29b* plasmid was introduced into *A. tumefaciens* strain GV3101 for transformation using Chinese cabbage C-24. The resulting OE Chinese cabbage transgenic plants were verified based on PCR amplification and GUS staining as described by Li et al. (2021).

### 2.9 Protein subcellular localization

To investigate the subcellular location of the transcription factor BrMYB29b, the coding sequence of BrMYB29b without the stop codon was amplified and cloned into the expression vector pCAMBIA 1302-GFP under the control of the CaMV 35S promoter. The subcellular localization of BrMYB29b proteins was detected by monitoring the green fluorescent protein (GFP) expression in *Arabidopsis thaliana* protoplast cells. *Arabidopsis* protoplast isolation, the transfection of plasmid DNA, and the protoplast culture protocol were performed with reference to Yoo et al. (2007). The fusion construct combined with nucleus localization protein (nls) was employed as a marker of the nucleus (Zhao et al., 2017).

### 2.10 Yeast one-hybrid assay

The yeast one-hybrid assay was performed as described in the Clontech PT3024-1/Yeast Protocols Handbook. *BrGSL-OHa* promoters obtained from the genomic DNA of Chiifu (1.88 kb) and RCBr (1.83 kb) were inserted into the pHIS2 plasmid. The full-length *BrMYB28a*, *BrMYB28b*, *BrMYB28c*, *BrMYB29a*, and *BrMYB29b* open reading frames were amplified from Chiifu cDNA and cloned into the pGADT7 vector (Clontech). The yeast transformants were selected on SD/−Trp/−Leu/−His medium and tested on SD/−Trp/−Leu/−His medium supplemented with various concentrations of 3-amino-1,2,4-triazole for 3 days at 30 °C.

### 2.11 Analysis of promoter cis-element interaction using a dual-luciferase reporter system

The full-length open reading frames of *BrMYB28a*, *BrMYB28b*, *BrMYB28c*, *BrMYB29a*, and *BrMYB29b* were cloned into the effector vector pCAMBIA1301S under the control of the CaMV 35S promoter. Two *BrGSL-OHa* promoter sequences obtained from the genomic DNA of Chiifu and RCBr were digested with *Hind*II and *BamH*I and inserted into the reporter vector pGreenII0800-LUC. These plasmids were co-transformed into *A. tumefaciens* strain GV3101 and used to infiltrate 1-month-old *Nicotiana benthamiana* leaves. The transient expression was analyzed following a 3-day incubation. The transcriptional activity in the infiltrated tobacco plants indicated by the ratio of firefly luciferase (LUC) to *Renilla* luciferase (REN) was assayed using a dual-luciferase reporter assay kit (Promega).

## 3 Results

### 3.1 Quantitative variation of leaf GSL content in the CRF_2/3_ population

A total of 13 GSLs were detected in the two parental materials and lines of the CRF_2/3_ population (**Supplementary Table S5**). The parental lines showed distinct GSL profiles in the 2012SG (spring, glasshouse) and 2013AF (autumn, open field) trials. GNA, a member of the aliphatic four-carbon group (Ali-4C), was the dominant GSL in the parent RCBr, with a maximum concentration of 20.76 µmol g^−1^ DW detected in 2013AF. In contrast, PRO, the hydroxylated product of GNA, was the dominant GSL in the parental line Chiifu, with a maximum concentration of 2.37 µmol g^−1^ DW observed in 2012SG. The concentrations of other Ali-GSL, Ind-GSL, and Aro-GSL compounds were generally higher in Chiifu than in RCBr but accounted for a small proportion of the total GSLs detected in both parents (**Supplementary Table S5**; **Supplementary Figure S1**).

In the CRF_3_ families, the contents of most individual GSLs and those of the five GSL types displayed a continuous distribution and wide variation (**Supplementary Table S5**), which suggests that the biosynthesis of each GSL is controlled by multiple genes in *B. rapa*. The CRF_3_ population exhibited transgressive segregation to a higher degree for most GSLs. Although the contents of glucoraphanin (GRA), glucobrassicanapin (GBN), glucobrassicin (GBS), and gluconasturtin (GNT) were relatively low in the parents, extremely high values were detected in the CRF_3_ family (**Supplementary Table S5**). As the dominant GSL compound type, Ali-GSLs contributed 56.67 and 50.51% of the total GSL content on average in the first and second trial, respectively, ranging from 5.41–91.84% and from 8.69–96.22%, respectively. Among Ind-GSLs, GBS, methoxy glucobrassicin (4ME), and neoglucobrassicin (NEO) contributed approximately 7–15% of the total GSL content in the CRF_3_ population, whereas 4-hydroxy glucobrassicin (4OH) made up a small percentage (0.41% in 2012SG and 0.33% in 2013AF). The significant variation in the content of each GSL in the mapping population facilitated subsequent QTL analysis.

### 3.2 QTL identification for GSL content in leaves

**Figure 1:**
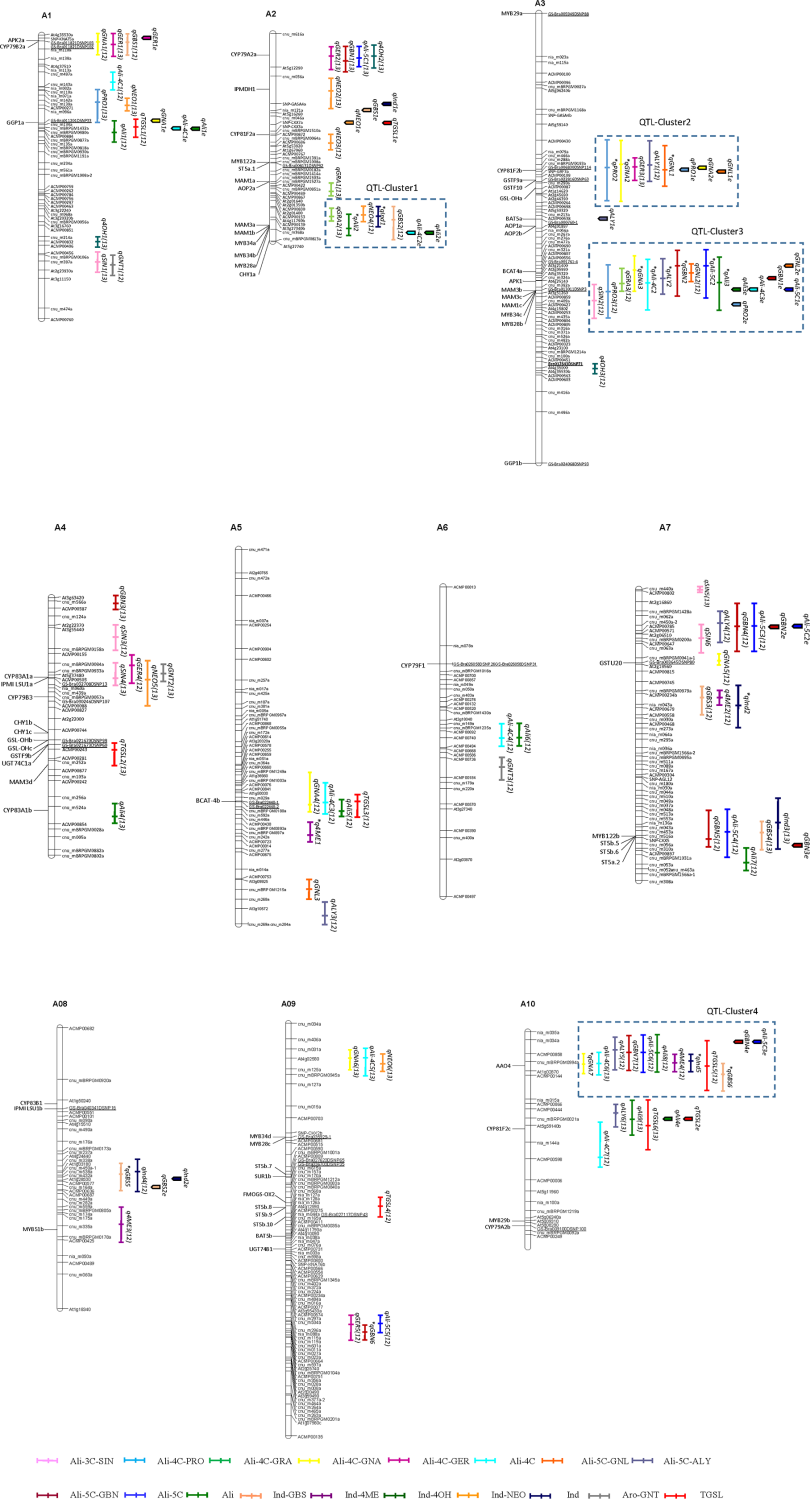
Distribution of quantitative trait loci (QTLs) for glucosinolate compounds in the *Brassica rapa* genome detected using the CRF_2/3_ bi-parental mapping population. QTL names are indicated by the abbreviations of individual glucosinolate names as shown in **Supplementary Table S5**. Numbers in parenthesis indicate the year of QTL detection. The asterisk before the name of each QTL represents a QTL detected in both trial years. Glucosinolate biosynthesis candidate genes anchored in the QTL region are shown on the left of each chromosome according to Wang et al. (2011a).

To investigate the genetic control of GSL biosynthesis in *B. rapa* leaves, a traditional QTL analysis was performed for 13 individual GSLs and five types of GSLs as well as the total GSL content in the CRF_2/3_ bi-parental mapping population in two different seasons and locations. A total of 60 QTLs for Ali-GSLs (Ali-QTLs) were identified on all chromosomes, with major loci detected on chromosomes A02 and A03 (**Figure 1**; **Supplementary Table S6**). Of the 60 Ali-QTLs, 12 were detected in both environments, while the remaining loci were detected in only a single environment. For the predominant GSL, GNA, seven QTLs were mapped, which explained 30.6 and 31.5% of the phenotypic variation in 2012AF and 2013SG, respectively. Three of these QTLs, namely, qGNA2 and qGNA3 on A03 and qGNA7 on A10, were identified in both environments. A major QTL cluster (QTL-Cluster3) on the middle-bottom of A03 was identified for gluconapin (qGNA3), glucoraphanin (qGRA3), progoitrin (qPRO3), a four-carbon side chain Ali-GSL (qAli-4C2), glucoalyssin (qALY2), glucobrassicanapin (qGBN2), gluconapoleiferin (qGNL2), a five-carbon side chain Ali-GSL (qAli-5C2), and the total Ali-GSLs (qAli3) in the two environments (2012AF and 2013SG), which accounted for 4–21.5% of the phenotypic variation (**Figure 1**; **Supplementary Table S6**). According to the physical positions of genetic markers and predicted GSL homologous genes in the *B. rapa* genome (Wang et al., 2011a), seven genes involved in Ali-GSL biosynthesis and regulation named *BrBCAT4a*, *BrAPK1*, *BrMAM3b*, *BrMAM3c*, *BrMAM1c*, *BrMYB34c*, and *BrMYB28b* were anchored in QTL-Cluster3. An additional region on A03 spanning from 50.4 to 74.8 cM (QTL-Cluster2) harbored a co-localized QTL for PRO, GNA, GER, ALY, and GNL that explained 5–20% of the phenotypic variation. Moreover, *BrGSL-OHa*, which is orthologous to an *Arabidopsis* 2ODD gene responsible for PRO formation, was found to be anchored in this region. The RCBr allele for the *BrGSL-OHa* locus increased the GNA content but decreased the PRO content. QTL-Cluster1, as a chromosome segment paralogous to the QTL-Cluster3 region, harbored not only Ali-QTLs (qGRA2 and qAli2) but also Ind-QTLs (qNEO4, qInd1, and qGBS2). QTL-Cluster 4 harbored both Ali-QTLs and Ind-QTLs. Of these QTLs, qGNA7 and qGBS6 were detected in the two trials, while the other QTLs were detected in one environment only (**Figure 1**; **Supplementary Table S6**).

The GenStat system was employed to detect QTLs affected by the environment. A total of 29 QTLs were obtained for 13 GSL phenotypes. Except for SIN, GRA, 4OH, and GNT, these QTLs were distributed on chromosomes A01, A02, A03, A07, A08, and A10 (**Figure 1**; **Supplementary Table S7**). Among them, six QTLs for six types of GSL displayed significant QTL–environment interactions (Q×E), reflecting the genetic variation underlying their phenotypic plasticity. qGER1e, qGBN4e, qAli-5C3e, and qNEO1e exhibited Q×E effects and were co-localized with their corresponding QTLs only in the 2012SG trial (**Figure 1**). qPRO1e, qGNA2e, and qGNL1e exhibited no Q×E effects and were mapped to QTL-Cluster2, in which QTLs were detected for PRO, GNA, and GNL in both the 2012SG and 2013AF trials (**Figure 1**). Four additional QTLs that displayed no interaction effects with the environment (qAli3e, qAli-4C3e, qGBN1e, and qAli-5C1e) were mapped to the same region of QTL-Cluster 3 on chromosome A03 (**Figure 1**).

### 3.3 Expression variant of *BrGSL-OHa* contributes to the QTLs for GNA and PRO compounds

**Figure 2:**
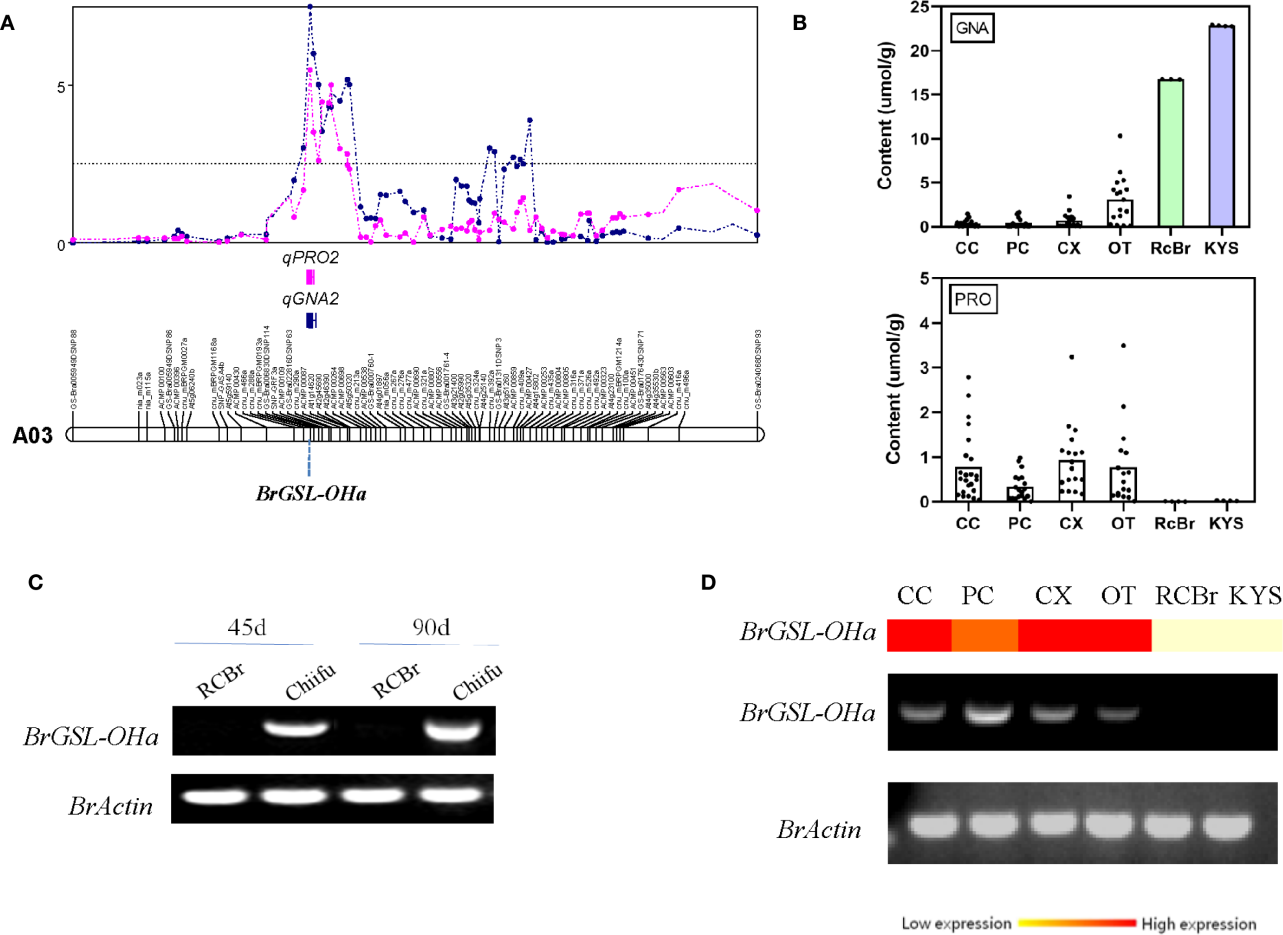
Quantitative trait locus (QTL) mapping for progoitrin and the expression of the candidate gene *BrGSL-OHa*. **(A)** QTL positions for progoitrin and gluconapin on chromosome A03. **(B)** Distribution of gluconapin (GNA) and progoitrin (PRO) contents among different types of *Brassica rapa*. CC, Chinese cabbage; PC, Pakchoi; CX, Caixin; OT, Oil-type. Rapid-cycling *B. rapa* (RCBr) and ‘KYS’ are two oil-type *B. rapa* varieties showing extremely high GNA content and low PRO content. **(C)** Semi-quantitative real-time polymerase chain reaction (RT-PCR) analysis of the candidate gene *BrGSL-OHa* using 46-day-old and 90-day-old fresh leaves of ‘Chiifu’ and RCBr. The *B. rapa* actin gene was used as a control for RNA quantification. **(D)** Real-time and semi-quantitative PCR analysis of *BrGSL-OHa* among lines representing different types of *B. rapa*.

Compared with the vegetable-type *B. rapa* lines, the oil-type RCBr line exhibited an extreme phenotype, with high GNA content and extremely low PRO content (**Supplementary Table S5**; **Supplementary Figure S1**). In the QTL-Cluster2 region, *BrGSL-OHa* (*Bra022920*) displayed the highest amino acid sequence similarity to the *Arabidopsis* 2ODD gene, which is responsible for PRO transformation from GNA (**Figure 2A**; **Supplementary Figure S2**). Genomic sequencing and PCR cloning results revealed that no significant differences were detected in the *BrGSL-OHa* coding sequences among *B. rapa*. Therefore, this study compared the transcript level of *BrGSL-OHa* among the two parents of the CRF_2/3_ mapping population and a small number of selected lines representing different types of *B. rapa*. *BrGSL-OHa* showed a higher transcript level in the parent Chiifu, whereas no transcripts were detected in RCBr (**Figure 2C**). Among the diverse *B. rapa* genetic lines, *BrGSL-OHa* transcripts were not detected only in the oil-type line ‘KYS’, which also showed an extremely high GNA content but less PRO (**Figures 2B, D**). Based on these results, it was predicted that *BrGSL-OHa* of the oil-type RCBr and KYS was a rare non-functional allele among *B. rapa* species, which resulted in an inability to synthesize PRO and thus the accumulation of high GNA content.

**Figure 3:**
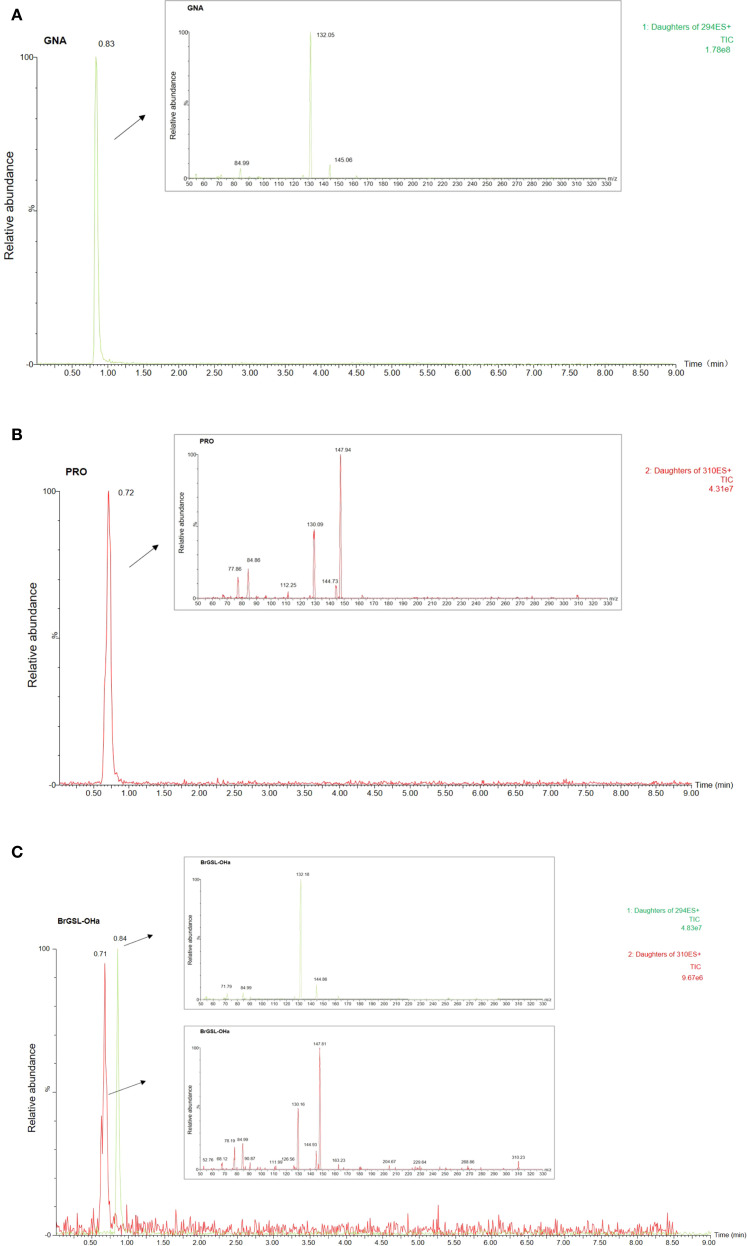
Enzymatic activity of BrGSL-OHa heterologously expressed in *Escherichia coli*. Total ion chromatography (TIC) and product ion spectrum scanning image of **(A)** desulfated gluconapin (GNA) standard, **(B)** desulfated progoitrin (PRO) standard, and **(C)** desulfoglucosinolates from a sample of GNA treated with heterologously expressed BrGSL-OHa fusion protein. The x-axis of the TIC image represents the retention time and the y-axis represents the relative abundance of ions. The mother ions of GNA (294) and PRO (310) are labeled on the right side of the TIC image. Arrows indicate the product ion spectrum scanning results for the targeted peak in the TIC image.

**Figure 4:**
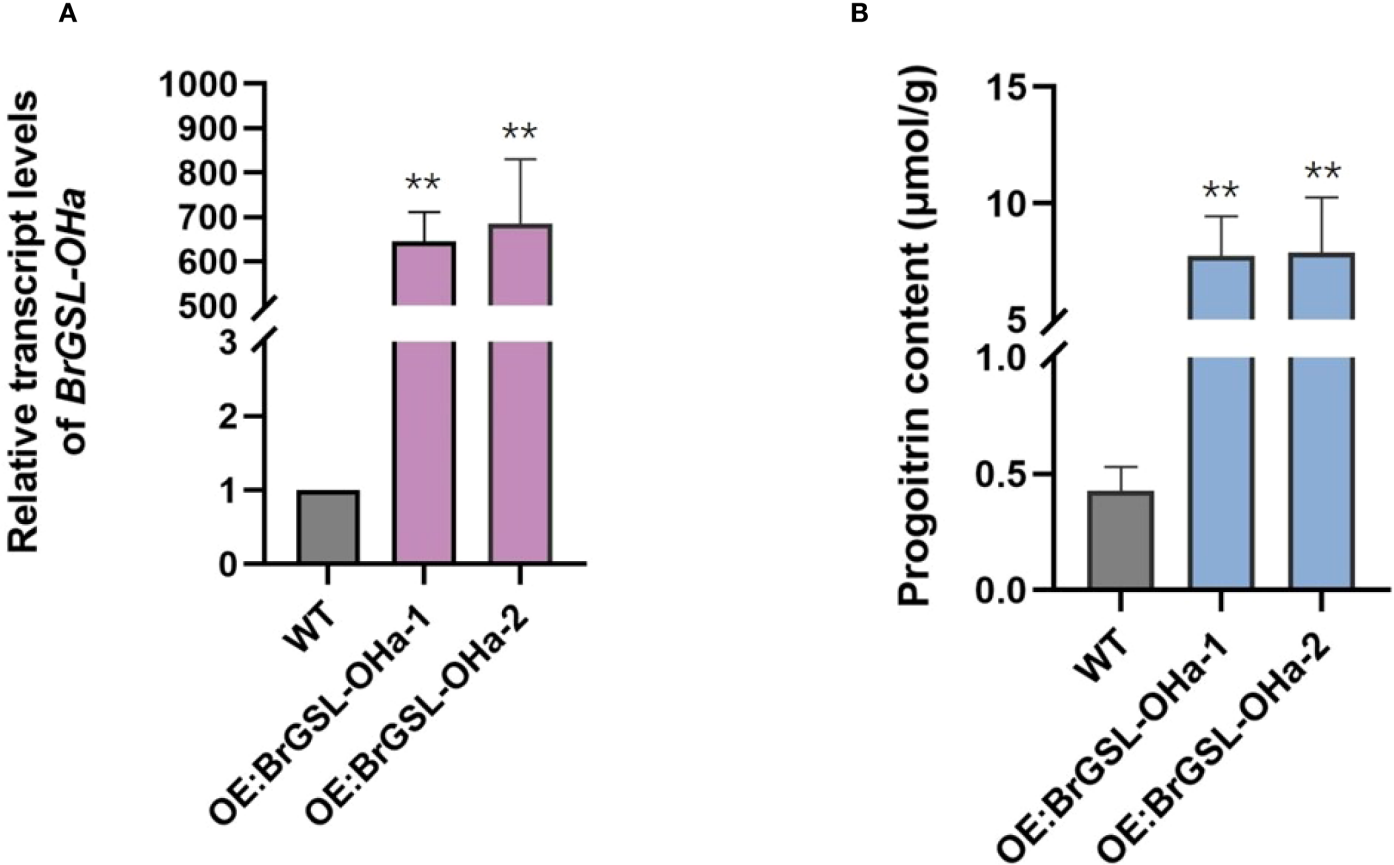
*BrGSL-OHa* confers progoitrin biosynthesis in *Brassica rapa*. Relative *BrGSL-OHa* expression levels **(A)** and progoitrin content **(B)** in leaves from overexpression transgenic plants (OE) and wild-type Chinese cabbage C-24 (WT) The data are presented as means ± SE from three independent biological replications. Black asterisks (**) indicate a significant difference (t-test, p < 0.01).

To validate the function of the predicted *BrGSL-OHa* for PRO biosynthesis from GNA in *B. rapa*, BrGSL-OHa protein was firstly heterologously expressed and purified in *E. coli* for enzyme catalytic activity assays (**Supplementary Figure S3**). As shown in **Figure 3**, the identities of two compounds in the sample containing GNA as substrate treated with BrGSL-OHa protein were confirmed by comparing their liquid chromatography–mass spectrometry profiles with those of GNA (**Figure 3A**) and PRO standards (**Figure 3B**). This indicated that BrGSL-OHa protein successfully catalyzed the conversion of GNA to PRO. Furthermore, *BrGSL-OHa* OE transgenic lines were generated for the Chinese cabbage C-24 background (**Supplementary Figure S4**). Compared with the wild-type C-24, two OE transgenic lines exhibited enhanced *BrGSL-OHa* expression levels accompanied by increased PRO content (**Figures 4A, B**), indicating that PRO biosynthesis was controlled by *BrGSL-OHa* expression.

### 3.4 Promoter variation of *BrGSL-OHa* contributes to its expression and PRO synthesis from GNA

**Figure 5:**
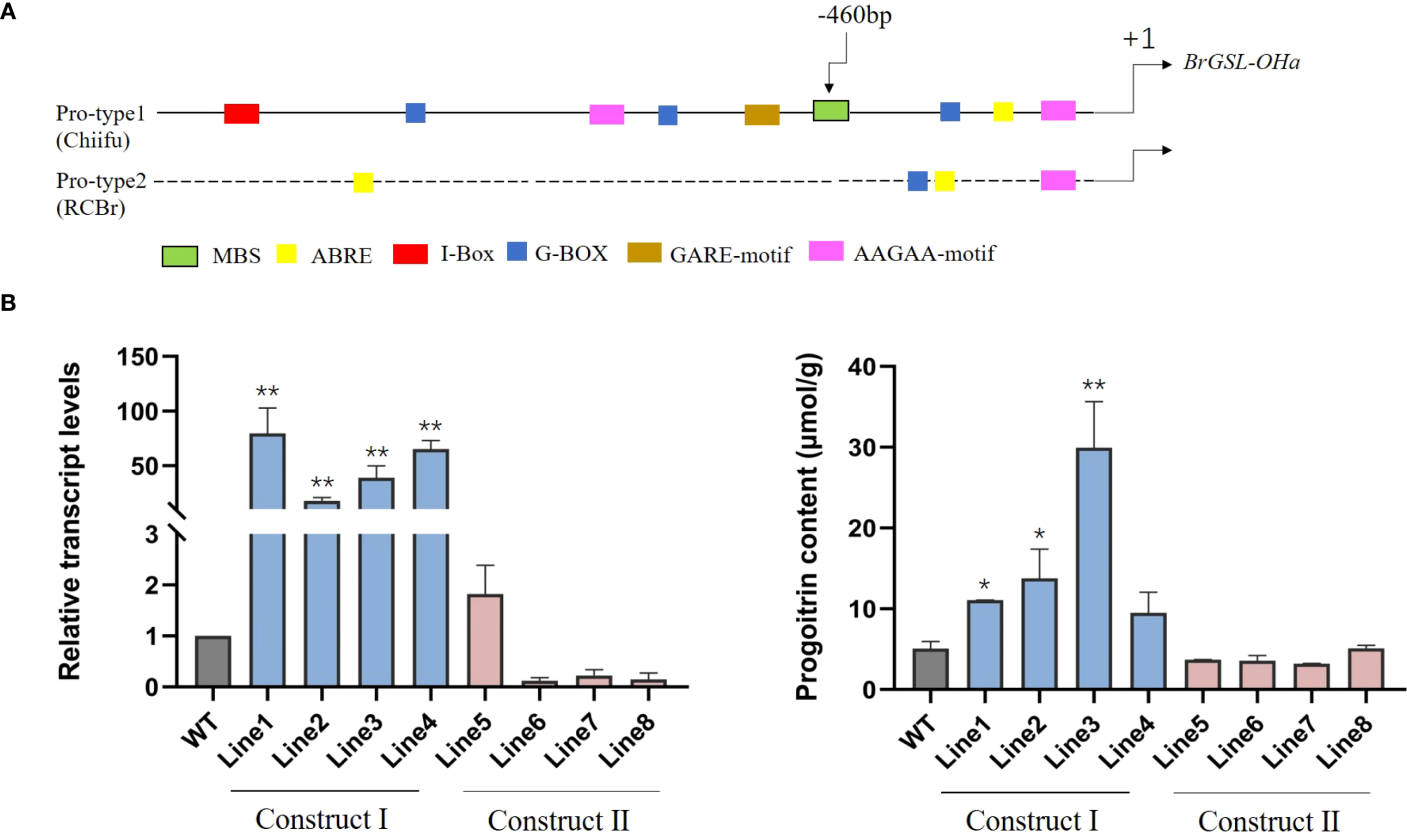
**(A)** Promoter sequence variation of *BrGSL-OHa* among *Brassica rapa* was comprised of two types: Pro-type1 was found in vegetable-type *B. rapa* and most oil-type *B. rapa*, as in ‘Chiifu’; and Pro-type 2 occurred in oil-type rapid-cycling *B. rapa* (RCBr). Predicted cis-elements are represented by colorful boxes. Relative transcript levels **(B)** and progoitrin content **(C)** in transgenic plants expressing construct I (Lines 1–4) and Construct II (Lines 5–8) compared with the wild-type Westar genotype (WT) The data are presented as means ± SE from three independent biological replications. Black asterisks (**) indicate a significant difference (t-test, p < 0.01), Black asterisks (*) indicate a significant difference (t-test, p < 0.05).

To determine whether the differential expression of *BrGSL-OHa* among two parental lines and *B. rapa* natural materials results from their promoter regions, this study cloned the promoter regions from Chiifu, RCBr, and 85 different *B. rapa* genotypes showing variation in GNA and PRO contents (**Supplementary Table S1**). The results revealed that the promoter sequences were highly conserved in *B. rapa*, including Chinese cabbage, pakchoi, caixin, and most oil-type *B. rapa* varieties, forming a Pro-type1 promoter, which was consistent with the Chiifu promoter sequence (**Figure 5A**). Considerable sequence differences were observed only in two oil-type *B. rapa* varieties, RCBr and KYS (**Figure 5A**). Promoter cis-element analysis demonstrated that an MYB binding site (MBS) was located at −460 bp in the Pro-type1 promoter of *BrGSL-OHa*, while only the RCBr and KYS varieties lacked the MBS (**Figure 5A**). The findings indicated that the absence of the MBS in the promoter regions of the oil-type *B. rapa* RCBr and KYS inhibited the expression of *BrGSL-OHa*, thereby blocking PRO synthesis and leading to the extreme accumulation of GNA.

To further demonstrate that variation in the *BrGSL-OHa* promoter alters its expression, this study generated transgenic plants using two types of promoters: *BrGSL-OHa* ^chiifupro:^ *BrGSL-OHa* ^chiifu^ (Construct I) and *BrGSL-OHa* ^RCBrpro:^ *BrGSL-OHa* ^RCBr^ (Construct II). As shown in **Figures 5B, C**, compared with the wild type, the transgenic lines expressing *BrGSL-OHa* driven by *BrGSL-OHa* ^chiifupro^ displayed significantly enhanced *BrGSL-OHa* transcript levels and progoitrin contents. In contrast, the transgenic lines expressing *BrGSL-OHa* ^RCBrpro:^ *BrGSL-OHa* ^RCBr^ did not accumulate PRO to the levels compared with that in the wild type. This result suggests a natural polymorphism in the promoter of *BrGSL-OHa* (the MBS deletion) is responsible for the gene expression.

### 3.5 *BrGSL-OHa* expression is directly regulated by the transcription factor BrMYB29b in *B. rapa*

**Figure 6:**
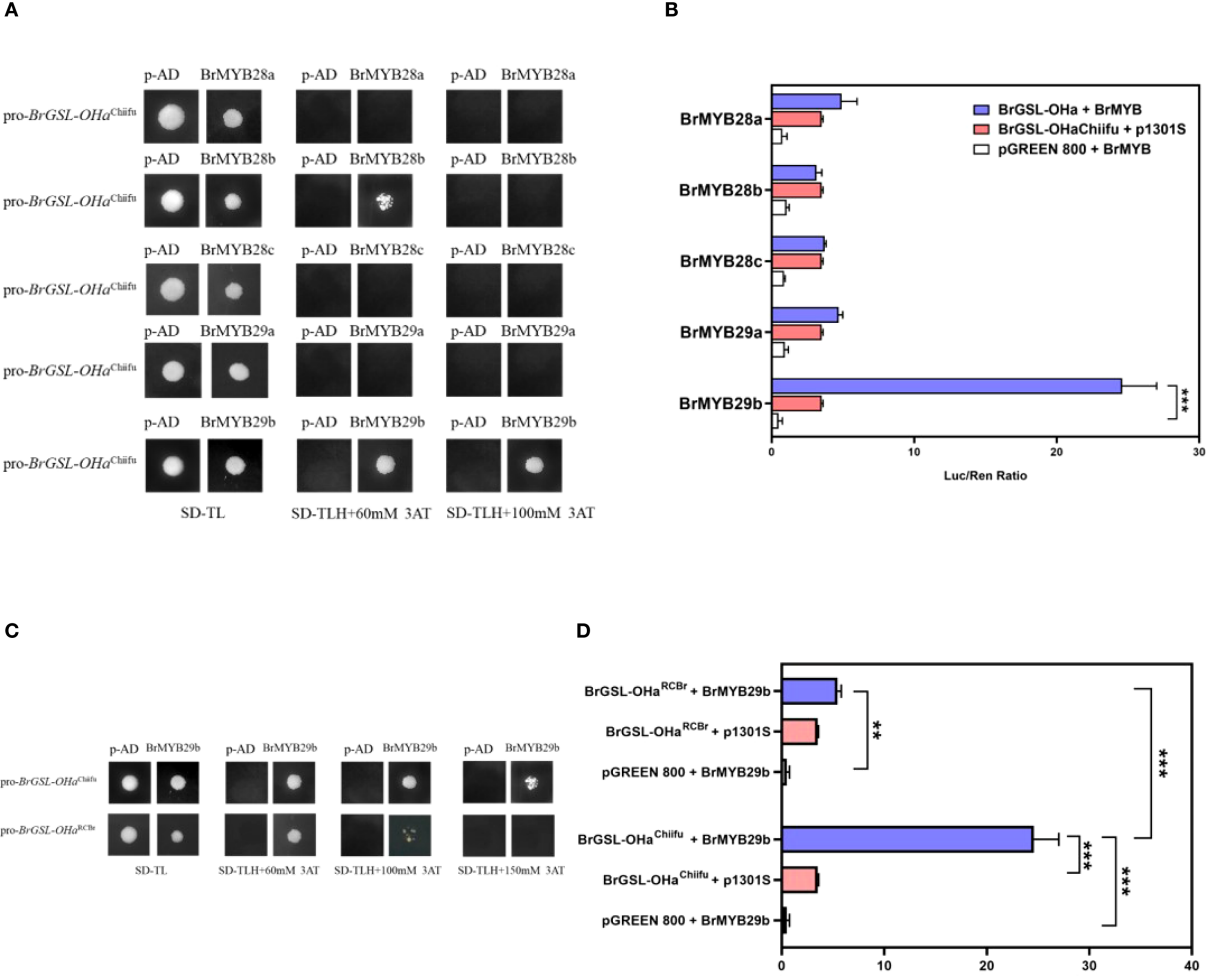
Identification of BrMYB29b as a transcription factor target of *BrGSL-OHa*. **(A)** Yeast-one hybrid (Y1H) assay of BrMYB29b binding to BrGSL-OHa^Chiifu^ promoter fragments among five MYB candidate genes related to glucosinolate (GSL) synthesis. **(B)** Dual luciferase system (LUC) detection of five MYB candidate genes binding to BrGSL-OHa^Chiifu^ promoter. The data are presented as means ± SE from three independent technical replications. Black asterisks (***) indicate a significant difference (t-test, p < 0.01), Black asterisks (**) indicate a significant difference (t-test, p < 0.05). **(C)** Y1H assay between BrMYB29b with BrGSL-OHaChiifu and BrGSL-OHa^RCBr^ promoter fragments. **(D)** Binding of BrMYB29b with the BrGSL-OHa^Chiifu^ and BrGSL-OHa^RCBr^ promoters according to the LUC assay results.

In *Arabidopsis*, three MYB transcription factors, MYB28, MYB29, and MYB76, have been shown to positively regulate a small number of structural genes in the Ali-GSL pathway (Gigolashvili et al., 2007a, 2007b, 2008; Hirai et al., 2007; Sonderby et al., 2010). In the *B. rapa* genome, three and two homologous genes to *AtMYB28* and *AtMYB29* have been identified, respectively, while no genes homologous to *AtMYB76* have been reported (Wang et al., 2011a, b; **Supplementary Table S2**). In the present study, one MBS was detected in the type 1 *BrGSL-OHa* promoter, but not in KYS and RCBr, two *B. rapa* oil-type genotypes (**Figure 5A**). According to the results of yeast one-hybrid and LUC analyses, BrMYB29b (Bra009245), which is homologous to AtMYB29, is a candidate transcription factor binding to cis-elements of the *BrGSL-OHa* ^Chiifu^ promoter (**Figures 6A, B**). Cells transformed with the *BrGSL-OHa* ^RCBr^ promoter lacking an MBS displayed less binding activity than cells transformed with the *BrGSL-OHa* ^Chiifu^ promoter, with an increasing 3-amino-1,2,4-triazole concentration (**Figure 6C**). To further improve this interaction, LUC reporter constructs were generated using two *BrGSL-OHa* promoter sequences from Chiifu and RCBr, which either contained or lacked an MBS, respectively. As shown in **Figure 6D**, the use of both the *BrGSL-OHa* ^Chiifu^ and *BrGSL-OHa* ^RCBr^ promoters could activate LUC signaling, but the *BrGSL-OHa* ^Chiifu^ promoter sequence was associated with significantly higher LUC activity than that of *BrGSL-OHa* ^RCBr^. These results suggest that BrMYB29b can bind directly to the *BrGSL-OHa* promoter and affect its expression. The absence of an MBS in the *BrGSL-OHa* promoter sequence of oil-type *B. rapa* influenced its ability to bind with BrMYB29b, thereby inhibiting its expression.

**Figure 7:**
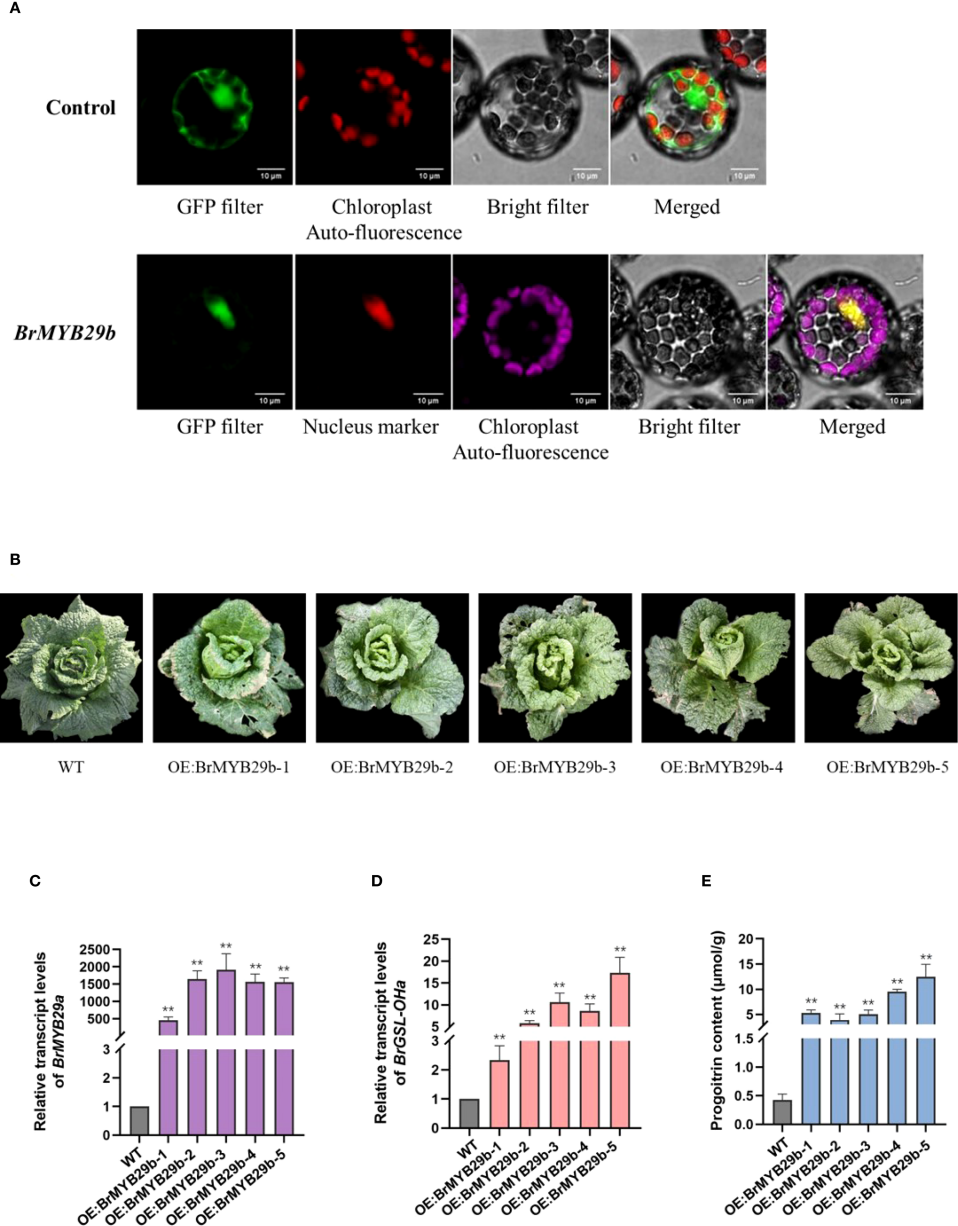
**(A)** Subcellular colocalization of transiently expressed BrMYB29b-GFP fusion protein with a nucleus marker (nls) in *Nicotiana benthamiana* leaves. **(B)** Phenotypes of the wild-type C-24 (WT) and *BrMYB29b*-overexpressing transgenic T_2_ plants. Relative transcript levels of *BrMYB29b* **(C)**, *BrGSL-OHa* **(D)**, and progoitrin content **(E)** in wild-type and *BrMYB29b*-overexpressing transgenic plants. The data are presented as means ± SE from three independent biological replications. Black asterisks (**) indicate a significant difference (t-test, p < 0.01).

To further validate the function of transcription factor BrMYB29b in *B. rapa*, this study first constructed BrMYB29b-GFP fusion protein to investigate its cellular localization. The BrMYB29b-GFP fluorescence signal overlapped with a marker for the nucleus (**Figure 7A**). This indicated that the BrMYB29b protein was located in the nucleus, conforming to the characteristics of transcription factors. Furthermore, this study constructed a BrMYB29b OE vector and transformed it into the Chinese cabbage C-24. In successfully transformed OE transgenic plants (**Figure 7B**), the BrMYB29b and *BrGSL-OHa* transcript levels as well as the PRO concentrations were markedly enhanced compared to the wild-type C-24 plants (**Figures 7C–E**). These results indicate that BrMYB29b is an important transcription factor responsible for positively regulating PRO biosynthesis in *B. rapa*. In addition, *BrMYB29b* OE plants displayed 1.5- to two-fold increases in the levels of GRA, GNA, and GBN compared to the wild type, while ALY and GNL did not increase significantly (**Supplementary Figure S5**). In addition to the *BrGSL-OHa* gene, the transcript levels of several key GSL biosynthesis-related genes including *MAM1* (*MAM1a*, *b*, *c*), *AOP2* (*AOP2a*, *b*, *c*), and *MYB28* (*MYB28a*, *b*, *c*) also increased (**Supplementary Figure S6**). In contrast, the expression level of another *MYB29* paralog (*MYB29a*, *Bra005949*) was not significantly different from that of the wild type. Taken together, these findings suggest that *BrMYB29b* can positively control Ali-GSL synthesis, with a particular effect on PRO content in *B. rapa*.

## 4 Discussion

GSL biosynthesis is an important but complex trait in *Brassica* crops. Our study revealed extensive variation in GSL compounds among *B. rapa* varieties. Three Ali-GSLs, namely, PRO, GNA, and GBN, are dominant compounds in *B. rapa* leaves, which has been reported previously (Lou et al., 2008; Kim et al., 2010). RCBr plants displayed extremely high GNA content and lower PRO content compared with Chiifu Chinese cabbage. Our discovery of a major QTL cluster on chromosome A03 anchored with tandem MAM genes and MYB transcription factors controlling Ali-GSLs is consistent with meta-QTL analyses in *B. oleracea*, which also identified conserved hotspots on chromosomes homologous to *A. thaliana* chromosome 5 (Sotelo et al., 2014). Similarly, in *B. napus*, GWAS studies have repeatedly identified the *BnaA03.MYB28* locus as a major determinant of both leaf and seed GSL content (Liu et al., 2020; Li et al., 2014), underscoring the conserved role of this genomic region across the Brassica genus. Four QTLs without Q x E effects identified using the multi-environment method were co-localized with QTL-Cluster3 for GNL, Ali-4C, GBN, and Ali-5C, suggesting that these QTLs play a stable genetic role and influence the earlier steps of the Ali-GSL biosynthesis pathway in *B. rapa*. Recently, Zhang et al. (2018) identified a transposon insertion of *BrMAM-3* (*Bra013007*) in the same QTL region associated with total Ali-GSLs. Further fine-scale mapping of this QTL region, expression profiling, and the functional validation of candidate genes are necessary.

Numerous genes involved in GSL biosynthesis have been identified and characterized in *Arabidopsis* (Kliebenstein et al., 2001b, c; Sonderby et al., 2010). Due to ancestral genome triplication, the B. rapa genome contains duplicated or triplicated homologs of many Arabidopsis genes, including those regulating GSL synthesis (Wang et al., 2011b). This genomic complexity has resulted in multiple copies of most GSL biosynthetic genes (Wang et al., 2011a), complicating the identification of key genetic determinants underlying GSL variation. In *Arabidopsis*, MYB28 is a central transcription factor regulating aliphatic GSL biosynthesis (Gigolashvili et al., 2007b). Here, we found that two *B. rapa* homologs, *BrMYB28a* and *BrMYB28b*, colocalized with QTL-Cluster1 and QTL-Cluster3, respectively, suggesting their potential role in controlling aliphatic GSL variation. Similarly, in *B. napus*, four MYB28 paralogs are located near GWAS peaks for seed GSL content (Li et al., 2014), and BnaMYB28 on chromosome A03 has been associated with high leaf but low seed GSL accumulation (Liu et al., 2020). While AtMYB28 directly activates genes such as *MAM*, *CYP79F1*, and *CYP83A1*, functional studies in *B. rapa* suggest that BrMYB28 paralogs may act as negative regulators of *AOP2* and positive regulators of *GSL-OH*—highlighting potential functional divergence from the Arabidopsis model. Thus, further investigation is needed to clarify the regulatory roles of different BrMYB28 paralogs in the GSL pathway.

**Figure 8:**
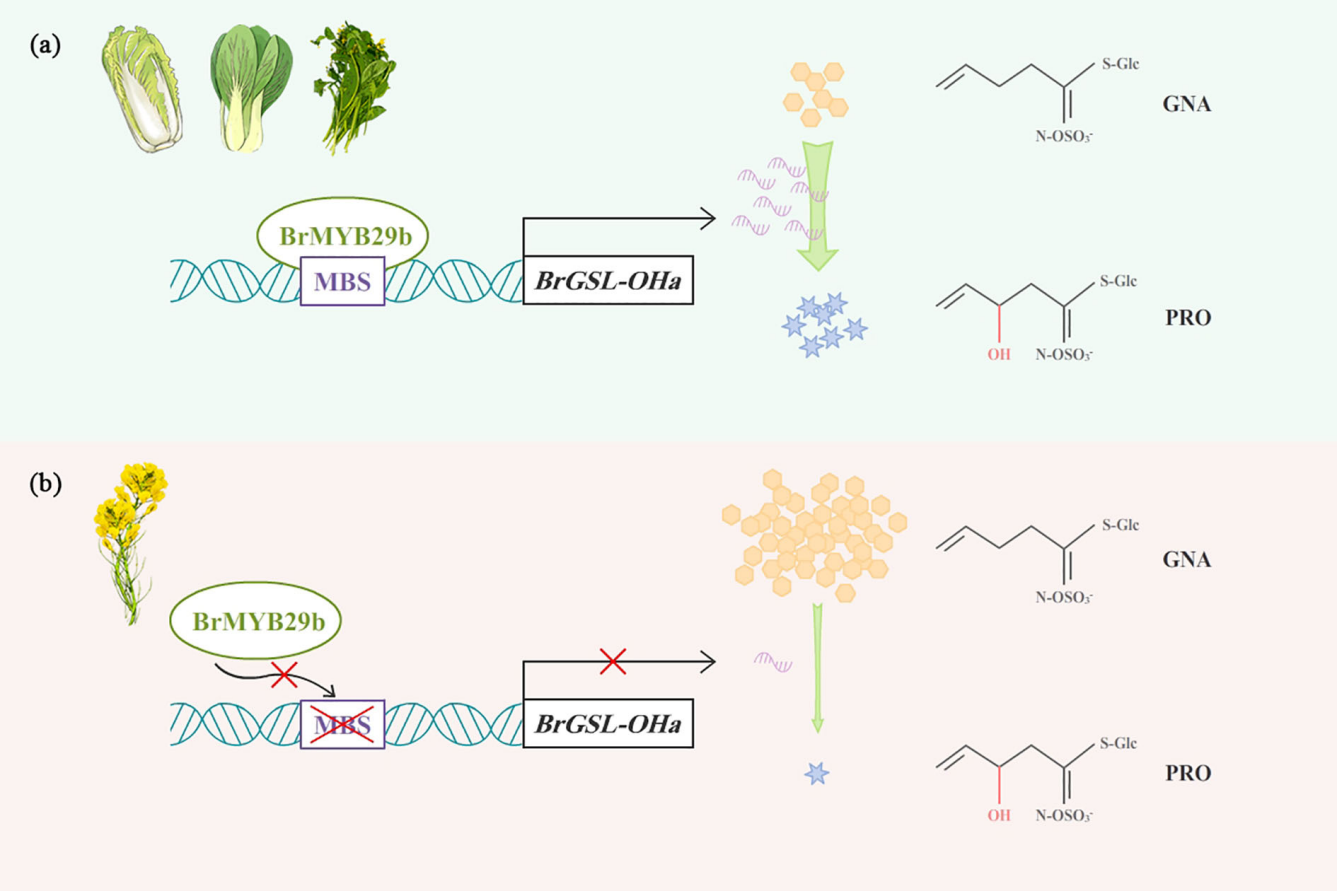
A proposed model for BrMYB29b binding to *BrGSL-OHa* promoter to regulate PRO biosynthesis in *B. rapa*. **(A)** In vegetable and most oil type *B. rapa*, BrMYB29b interacted with *BrGSL-OHa* promoter and leads to high expression of *BrGSL-OHa* that promotes PRO synthesis. **(B)** In two oil-type *B. rapa*, RCBr and KYS, lack of MBS in *BrGSL-OHa* promoter affects the binding ability with BrMYB29b, thus inhibiting *BrGSL-OHa* expression and PRO conversion from GNA.

In *Arabidopsis*, the *GSL-OH* gene was detected in a strong linkage disequilibrium region associated with PRO and GNL (Brachi et al., 2015) and exerted epistatic effects on fitness with the *MAM* locus. In the bi-parental population employed in this study, *BrGSL-OHa* anchored to the hot-spot QTL region controlling GNA, PRO, and GNL contents showed no sequence variation among natural population lines, while variation in its expression may explain this QTL. Further promoter sequence analysis indicated that the lack of an MBS in oil-type RCBr and KYS influenced the interaction between the transcription factor BrMYB29b and *BrGSL-OHa* and inhibited the expression of *BrGSL-OHa*, thereby blocking PRO biosynthesis and inducing the massive accumulation of GNA, as shown in the working model (**Figure 8**). In *Arabidopsis*, Hansen et al. (2008) cloned *GSL-OH* loci using fine-scale mapping and found that a repeated 120-bp motif in the *AtGSL-OH* promoter was related to its transcript and PRO content. This stark contrast highlights that while the function of the GSL-OH enzyme in catalyzing the conversion of GNA to PRO is conserved between *Arabidopsis* and *B. rapa* (Hansen et al., 2008), the transcriptional mechanisms governing the expression of their respective genes have diverged significantly, likely reflecting the distinct evolutionary and breeding histories of these species. *BrMYB29b* OE resulted in increased levels of GRA, GNA, and GBN, while the PRO content increased the most dramatically; this finding demonstrated that *BrMYB29b* positively regulated Ali-GSLs, particularly PRO, and enhanced the *BrGSL-OHa* transcription level in *B. rapa*. The results showed that three *B. rapa MYB28* paralogs (*MYB28a*, *b*, *c*) were all activated by the OE of *BrMYB29b*, which was probably caused by their interaction. Similarly, *BoMYB29(2)* OE in *B. oleracea* displayed up-regulated transcription levels of *BoGSL-OH(2)*, *BoAOP2(1)*, and *BoMYB28(3)*. In contrast, *Arabidopsis MYB29* was activated by *MYB28*, whereas *MYB28* was neither strongly activated nor repressed by *MYB29*. Additionally, the expression level of *BrMYB29a* paralog showed no change in *BrMYB29b* OE transgenic plants compared to the wild type (**Supplementary Figure S6**). This demonstrated that the paralog function of *BrMYB29a* was independent on *BrMYB29b.* It is speculated that *B. rapa MYB28/MYB29* paralogs exhibit functional differentiation in the GSL synthesis pathway and are probably able to activate different biosynthetic genes directly. Therefore, more work is needed to elucidate the functions and regulatory networks of these MYB transcription factors in *B. rapa*.

Beyond elucidating a key molecular mechanism controlling GSL variation, our discovery of a rare, non-functional *BrGSL-OHa* allele in specific oil-type lines provides insight into potential adaptive or human-directed selection. Progoitrin is an anti-nutritional compound that diminishes the quality of oilseed meal for animal feed. The MBS deletion, resulting in low PRO and high GNA accumulation, likely represents a desirable trait that was inadvertently or deliberately selected during the breeding of these specific oil-type lineages. This allele is not widespread even among oil-types, suggesting it is a valuable, niche genetic resource rather than a universal domestication trait. Its identification enables the use of marker-assisted selection to precisely introgress this allele into elite oilseed *B. rapa* cultivars, aiming to improve meal quality without compromising other agronomic traits. Conversely, in vegetable-type *B. rapa* where certain GSLs contribute to flavor and disease resistance, the functional Pro-type1 allele would be preferred. Therefore, understanding the lineage-specificity of this mutation empowers breeders to tailor glucosinolate profiles for different end uses, balancing defense, nutrition, and consumer preference.

## Data Availability

The original contributions presented in the study are included in the article/**Supplementary Material**. Further inquiries can be directed to the corresponding authors.
