# Supplementary material for: A naturally occurring promoter variation of BrGSL-OHa contributes to the conversion of gluconapin to progoitrin in Brassica rapa L. leaves
Source: Front Plant Sci. 2025 Sep 24;16:1654238. doi: 10.3389/fpls.2025.1654238 (PMC12504251; doi:10.3389/fpls.2025.1654238)
Supplement: Supplementary file 2 [file DataSheet1.docx]

Supplementary Material

# Supplementary Figures


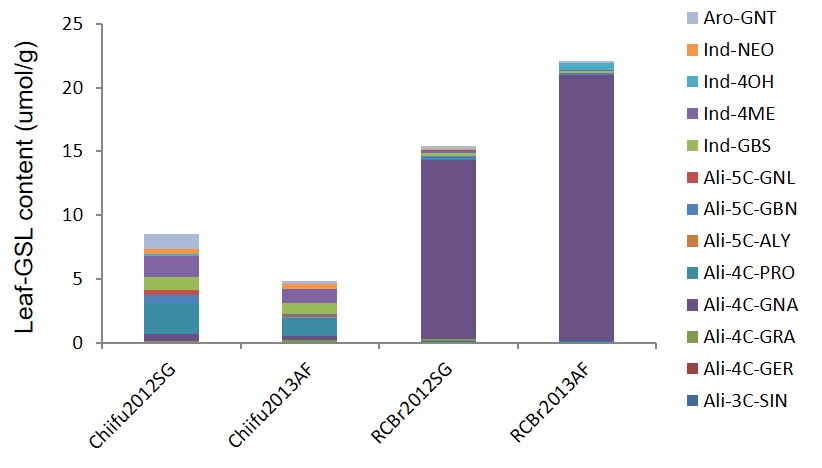


**Figure S1.** Distribution of glucosinolate content in the leaves of two parents of CRF_2/3_ population in 2012SG and 2013AF trials, respectively.


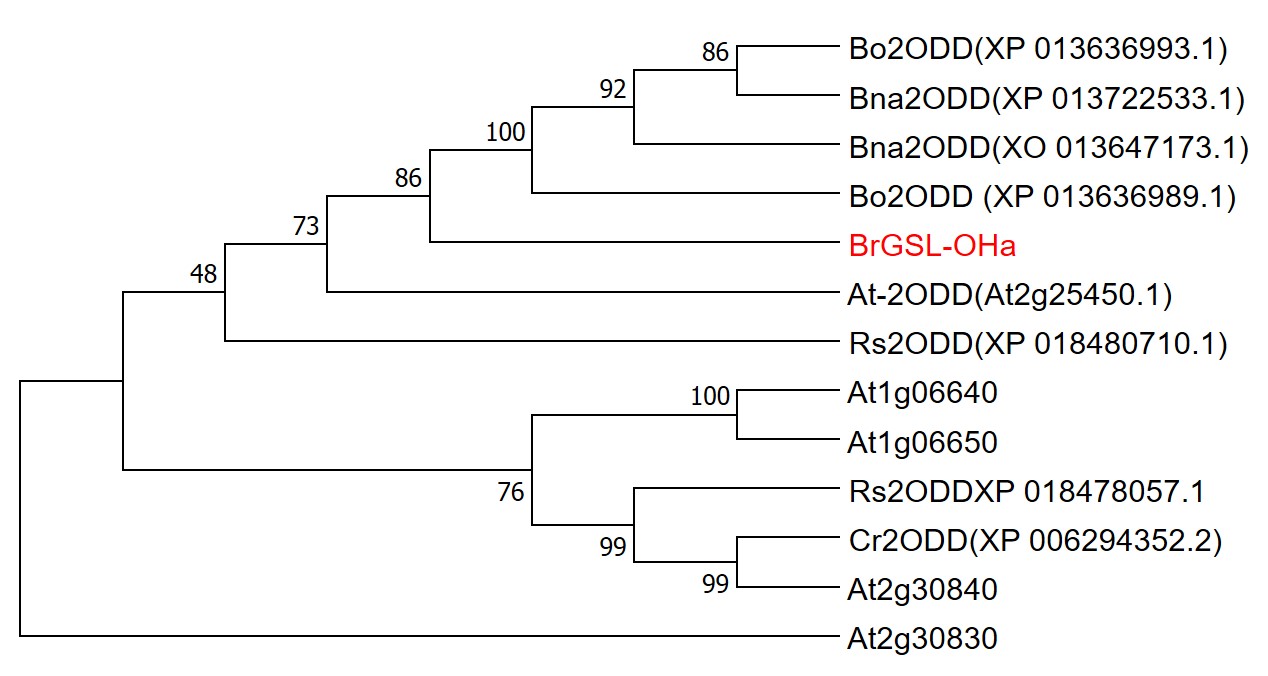


**Figure S2.** Phylogenetic analysis of BrGSL-OHa orthologs in Brassicaceae plants. Full-length protein sequences of BrGSL-OHa orthologs from different Brassiceaea plants were downloaded following NCBI-BLAST and TAIR. The phylogenetic tree was constructed using MEGA7.0 software using the neighbor-joining method in the bootstrap test (1000 replicates). The evolutionary distances were computed using the Poisson correction method and are in the units of the number of amino acid substitutions per siteBna, *Brassica napus*; Bo, *Brassica oleracea*; At, *Arabidopsis thaliana*; Rs, *Raphanus sativa*

**
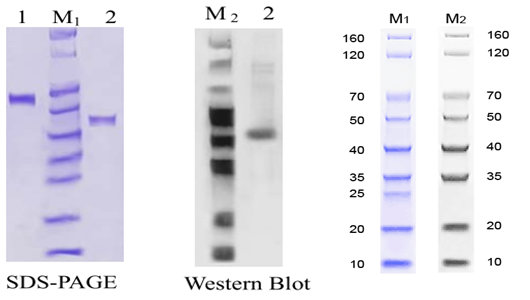
**

**Figure S3.** BrGSL-OHa protein expressed in Escherichia coli strain BL21(DE3). Lane 1: BSA (1.5ug); Lane 2: BrGSL-OHa protein (1.5ug); M1: SDS-PAGE Marker; M2: Western Blot Marker.


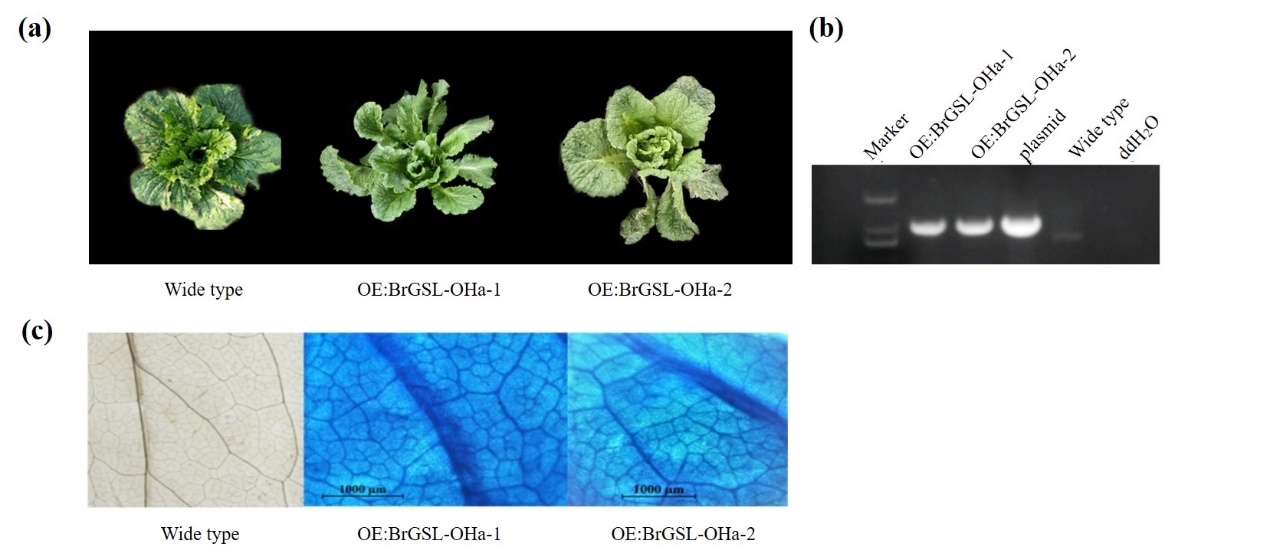


**Figure S4.** Phenotype (a), PCR detection (b) and GUS staining (c) of wild type C-24 Chinese cabbage and *BrGSL-OHa*-overexpression transgenic plants.


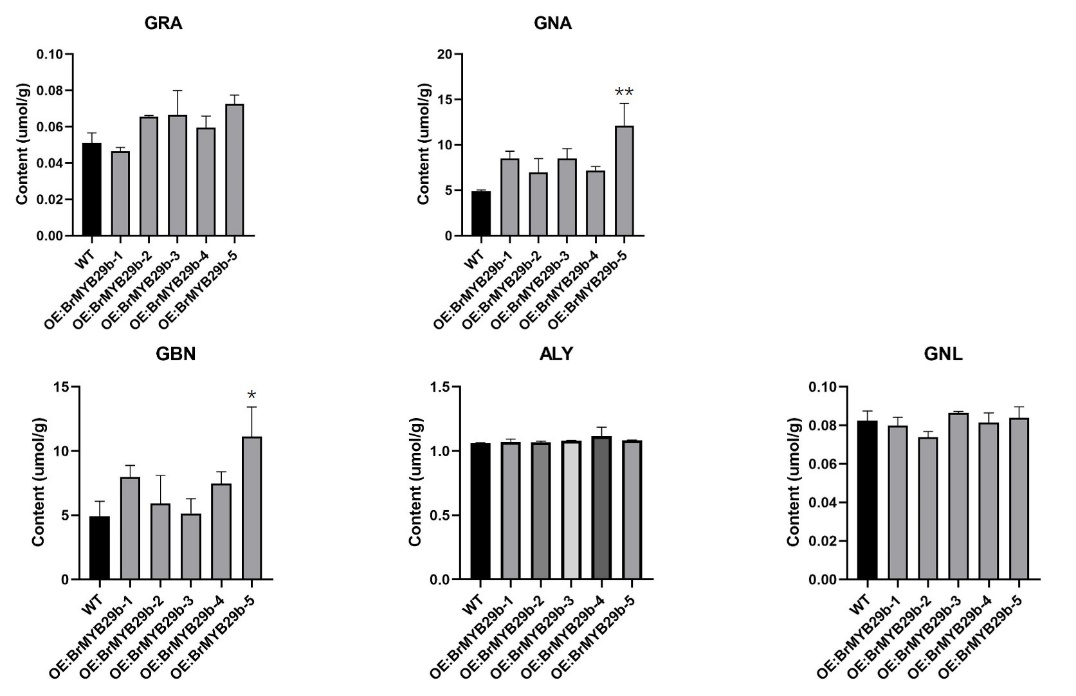


**Figure S5.** Individual aliphatic glucosinolate compounds in wild type and BrMYB29b-overexpression Chinese cabbage transgenic lines.


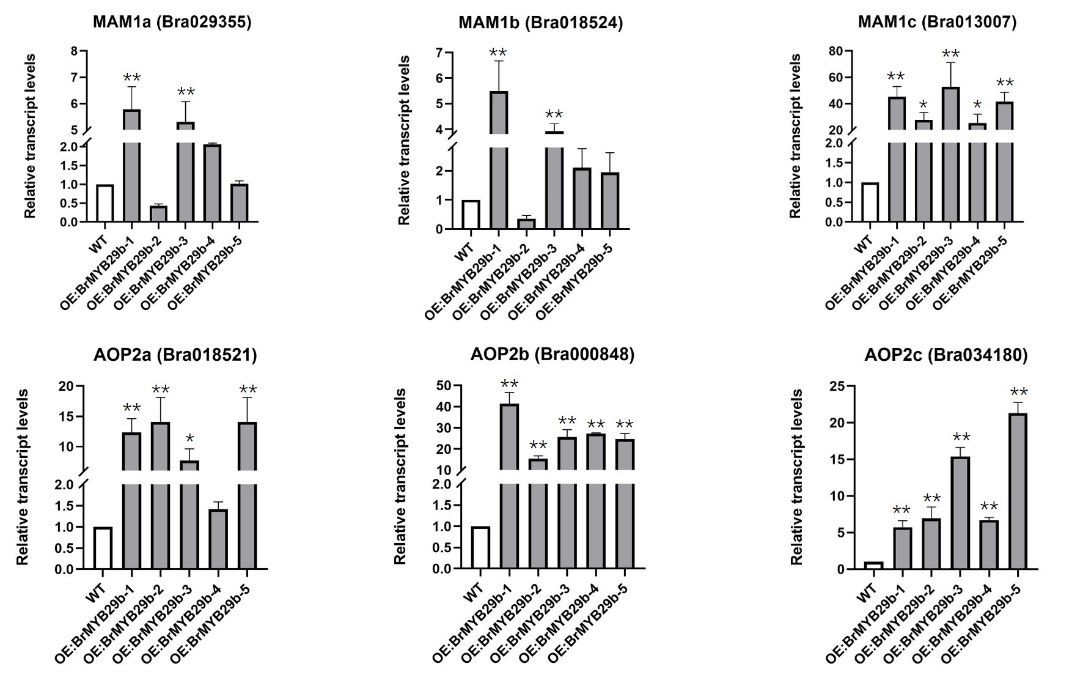


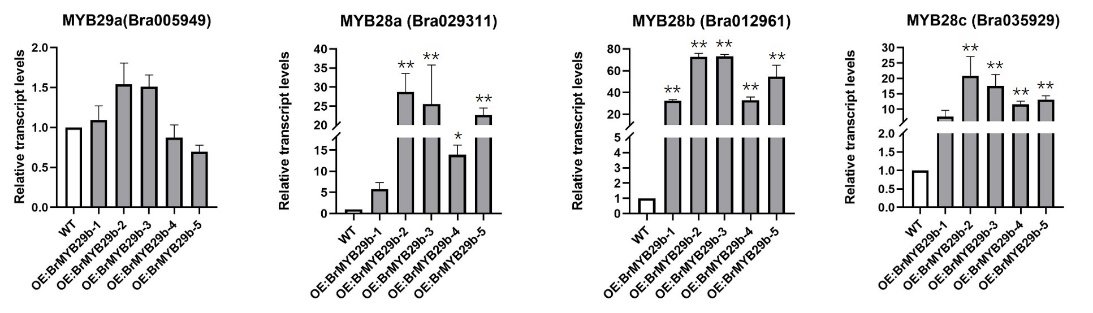


**Figure S6.** Relative transcript levels of aliphatic GSL related biosynthesis genes in wild type and BrMYB29b-overexpression Chinese cabbage transgenic lines.
